# An Implantable Vascularized Protein Gel Construct That Supports Human Fetal Hepatoblast Survival and Infection by Hepatitis C Virus in Mice

**DOI:** 10.1371/journal.pone.0009987

**Published:** 2010-04-01

**Authors:** Martha J. Harding, Christin M. Lepus, Thomas F. Gibson, Benjamin R. Shepherd, Scott A. Gerber, Morven Graham, Frank X. Paturzo, Christoph Rahner, Joseph A. Madri, Alfred L. M. Bothwell, Brett D. Lindenbach, Jordan S. Pober

**Affiliations:** 1 Section of Comparative Medicine, Yale University School of Medicine, New Haven, Connecticut, United States of America; 2 Department of Immunobiology, Yale University School of Medicine, New Haven, Connecticut, United States of America; 3 Center for Cellular and Molecular Imaging, Yale University School of Medicine, New Haven, Connecticut, United States of America; 4 Department of Pathology, Yale University School of Medicine, New Haven, Connecticut, United States of America; 5 Section of Microbial Pathogenesis, Yale University School of Medicine, New Haven, Connecticut, United States of America; 6 Department of Dermatology, Yale University School of Medicine, New Haven, Connecticut, United States of America; Institut Pasteur, France

## Abstract

**Background:**

Widely accessible small animal models suitable for the study of hepatitis C virus (HCV) *in vivo* are lacking, primarily because rodent hepatocytes cannot be productively infected and because human hepatocytes are not easily engrafted in immunodeficient mice.

**Methodology/Principal Findings:**

We report here on a novel approach for human hepatocyte engraftment that involves subcutaneous implantation of primary human fetal hepatoblasts (HFH) within a vascularized rat collagen type I/human fibronectin (rCI/hFN) gel containing Bcl-2-transduced human umbilical vein endothelial cells (Bcl-2-HUVEC) in severe combined immunodeficient X beige (SCID/bg) mice. Maturing hepatic epithelial cells in HFH/Bcl-2-HUVEC co-implants displayed endocytotic activity at the basolateral surface, canalicular microvilli and apical tight junctions between adjacent cells assessed by transmission electron microscopy. Some primary HFH, but not Huh-7.5 hepatoma cells, appeared to differentiate towards a cholangiocyte lineage within the gels, based on histological appearance and cytokeratin 7 (CK7) mRNA and protein expression. Levels of human albumin and hepatic nuclear factor 4α (HNF4α) mRNA expression in gel implants and plasma human albumin levels in mice engrafted with HFH and Bcl-2-HUVEC were somewhat enhanced by including murine liver-like basement membrane (mLBM) components and/or hepatocyte growth factor (HGF)-HUVEC within the gel matrix. Following *ex vivo* viral adsorption, both HFH/Bcl-2-HUVEC and Huh-7.5/Bcl-2-HUVEC co-implants sustained HCV Jc1 infection for at least 2 weeks *in vivo*, based on qRT-PCR and immunoelectron microscopic (IEM) analyses of gel tissue.

**Conclusion/Significance:**

The system described here thus provides the basis for a simple and robust small animal model of HFH engraftment that is applicable to the study of HCV infections *in vivo*.

## Introduction

Hepatitis C virus (HCV) is a major cause of morbidity and mortality, resulting in hepatitis, cirrhosis and liver failure, hepatocellular carcinoma and immune complex vasculitis. Because HCV replicates efficiently only in human or chimpanzee hepatocytes, there is no natural small animal model to study HCV infection *in vivo*. The most successful mouse model to date uses albumin promoter-urokinase-type plasminogen activator (Alb-uPA)-transgenic mice that overexpress uPA in the liver which results in hypofibrinogenemia, neonatal bleeding and progressive liver degeneration [1]. Although this defect is lethal in homozygous mice, they can be rescued by transplanting wild-type murine or human hepatocytes within the first several weeks of life [2], [3]. In the latter system, levels of human hepatocyte chimerism can develop within the liver parenchyma of engrafted Alb-uPA mice sufficient to routinely sustain HCV infection for at least 4 months duration [4]–[6]. However, the system is difficult to work with since homozygote uPA mice invariably die as neonates if not engrafted with wild-type hepatocytes within 2–3 weeks of life [4], [5], [7]. Furthermore, engrafted Alb-uPA mice harboring high levels (>50%) of human hepatocytes within the liver parenchyma suffer from lethal human complement-mediated kidney damage within 3 months of life, if not treated continuously with anti-complement factor drugs [7]. Therefore, the development of strategies for human hepatocyte engraftment into healthy adult mice could significantly expand the accessibility and numbers of mice available to study human liver diseases, including HCV infections.

We report conditions for the efficient engraftment of human liver cells at a heterotopic site in adult recipient mice. A subcutaneous transplantation route was selected because of its simplicity as a surgical procedure, its provision of ample space for graft expansion compared to alternate sites, such as under the kidney capsule, and accessibility of the graft for diagnostic and experimental analyses by routine biopsy and noninvasive imaging. Transformed hepatoma cells have been shown to survive following subcutaneous injection in immunodeficient mice and provide a useful pre-clinical system for screening novel anti-HCV therapeutic agents [8]. A key characteristic of tumor cells that distinguishes them from normal cells is their proclivity to induce angiogenesis, a critical step in tumor development and growth [9]. Sustained maintenance of primary human hepatocytes following transplantation at subcutaneous sites has not yet been realized [10], [11]. We reasoned that, in contrast to hepatoma or other tumor cells, non-transformed hepatocytes are unable to promote angiogenesis, and as such, unable to induce the rapid neovascularization required to maintain a normoxic environment and hence cell viability. Our laboratory has previously shown that Bcl-2-transduced human umbilical vein endothelial cells (Bcl-2-HUVEC) are able to form a well-differentiated vascular network when implanted subcutaneously in protein gels in the severe combined immunodeficient X beige (SCID/bg) mouse strain [12]. Although this strain is less adept at accepting hematopoietic stem cell grafts than certain other immunodeficient mice [13], they are surprisingly superior to these newer strains at accepting vascularized grafts [14]. The goal of the present study was to utilize such vascularized gels to support subcutaneous hepatocellular engraftment and HCV infections *in vivo.*

## Methods

### Ethics statement

All experimentation in mice was approved by the Yale University Institutional Animal Care and Use Committee. The collection and work with HFH was approved by the Institutional Review Boards of Yale University and Albert Einstein College of Medicine (AECOM). Following written maternal consent, Human Fetal Tissue Repository (AECOM hFTR, Dr. Brad Poulos, Director) staff collected liver tissue from 14–18 week gestational age abortuses following elective terminations. HUVEC were harvested from discarded, anonymized umbilical cords with approval from the Institutional Review Board of Yale University. The collection of serum from patients with chronic HCV was approved by the Institutional Review Board of Yale University and was harvested following written patient consent.

### Mice

SCID/bg mice (C.B-17/GbmsTac-SCID/bgN7) were purchased from Taconic (Germantown, New York), housed in microisolator cages in the Yale Animal Resources Center and provided free access to autoclaved mouse chow (Harlan, Madison, WI) and autoclaved, hyperchlorinated water.

### HFH cells

Human fetal liver (HFL) tissue was transported to the laboratory (approximately 1.5 hours travel time) in ice-cold Hanks buffered salt solution (without calcium and magnesium; Invitrogen, Carlsbad, CA). HFL tissue was minced into small pieces, then cells harvested by digesting with 5 mg/ml collagenase type D (Roche, Indianapolis, IN) in buffer (per 100 ml: 0.39 g NaCl, 0.05 g KCl, 0.24 g HEPES, 0.07 g CaCl_2_.2H_2_0) at 37°C for 30 minutes with gentle agitation. Cells were washed in buffer at 50 x g for 5 minutes then EGTA buffer (5 mM EGTA in Hanks buffered salt solution, plus 1% BSA, 100 mM Hepes and 14 mM glucose). HFH were allowed to adsorb to hydrophilic tissue culture plates (Primaria, Becton Dickinson, San Jose, CA) or rat tail collagen I (rCI)-coated tissue culture plates (Beckton Dickinson) in Hepatocyte Culture Medium (HCM), composed of Williams E medium supplemented with 10 mM nicotimamide, 20 mM HEPES, 17 mM NaHCO_3_, 11 mg/ml pyruvate, 0.2 mM ascorbic acid, 14 mM glucose, 2 mM L-glutamine, 10^−7^ M dexamethasone, 6.26 ug/ml ITS premix (insulin, transferrin, selenious acid), 1.25 mg/ml bovine serum albumin, 5.35 µg/ml linoleic acid, 200 units/ml penicillin, 200 µg/ml streptomycin and 5.0 µg/ml amphotericin B [15]. Fetal calf serum (FCS; 5%) was added during the initial 24 hour attachment phase and then serum-containing medium was removed and replaced with medium containing 20 ng/ml epidermal growth factor (Invitrogen). Cells were expanded by culturing for up to 1 month with media changes every 2 days. To further expand cell numbers, cells were passaged by washing cultures twice with phosphate buffered saline (PBS; pH = 7.2), incubating cells with EGTA buffer for 5–10 minutes at 37°C, then rinsing in collagenase buffer. Cells were washed in EGTA as described above and flasks seeded in a 1∶2–1∶3 ratio or cells used immediately to prepare vascularized gels, as described below.

### Phenotyping of HFL cultures

Passage 1 HFL cells, harvested from a 15 week gestational age HFL, were grown for 3 days on coverslips coated with 1 mg/ml rCI (BD Biosciences, San Diego, CA), then fixed with 4% paraformaldehyde in PBS for 15 minutes at room temperature. Cells were permeabilized and blocked by treating with PBS containing 0.1% Tween-20 (PBS-T) plus 1% gelatin (for albumin detection) or PBS-T plus 1% BSA (for all other intracellular markers) for 30 minutes at room temperature. Cells were washed and incubated for 1 hour at room temperature in the above blocking solutions with the following primary mouse antibodies: anti-human albumin (Sigma, St. Louis, MO), anti-human cytokeratin (CK) 8/18 (Abcam, Cambridge, MA), anti-human CK19 (Chemicon, Temecula, CA), anti-human hepatocyte (HepPar1; DAKO, Carpinteria, CA), anti-human epithelial-specific antigen (HEA125; Progen Biotechnik, Heidelberg, Germany), and anti-α-smooth muscle actin (SMA; Novocastra, Norwell, MA). Alternatively, cells were stained with PE-conjugated anti-human VE-cadherin (R&D Systems, Minneapolis, MN) or PE-conjugated anti-human CD31 (Immunotech, Fullerton, CA). Unconjugated antibodies were detected with Cy3-labelled goat anti-mouse IgG (H+L) (Jackson ImmunoResearch Laboratories, West Grove, PA).

### Huh-7.5 cells

Huh-7.5 cells were a generous donation from Dr. Charles Rice, Rockefeller University. Cell cultures were grown on polystyrene tissue culture plastic-ware in DMEM plus 10% FCS, 2 mM L-glutamine, non-essential amino acids, 100 units/ml penicillin and 100 µg/ml streptomycin. Cells were passaged by using 0.25% trypsin/1 mM EDTA (Invitrogen) and seeded at a dilution of 1∶2–1∶3, or cells used immediately to prepare vascularized gels, as described below.

### Preparation of retrovirally transduced HUVEC

HUVEC were derived from discarded, anonymized human tissues as described [16], [17]. Cultures were transduced with a retrovirus (LZRS-pBMN-Z, a gift from Dr. Garry Nolan, Stanford University) encoding caspase-resistant Bcl-2 ([18] kindly provided by Dr. Marie Hardwick, Johns Hopkins School of Public Health, Baltimore, MD) and generated as described [19]. An additional retrovirus encoding the deletion variant (i.e. highly active) form of hepatocyte growth factor (HGF) [20] was prepared by subcloning the HGF gene from HGF-pcDNA3.1 ([21], a generous donation from Dr. Youhua Liu, University of Pittsburgh) into LZRS-pBMN-Z at BamHI and NotI sites, following PCR amplification of the HGF gene by using the following primers (forward: 5′-AGACATGGATCCATAAGAAAGCGGCCATGTGGGTGACCAAACTCCTG-3′; adds a BamHI site) and (reverse: 5′-ATAGTTTAGCGGCCGCATTCTTATCTATGACTGTGGTACCTTATA-3′; adds a NotI site). The production of HGF by HGF-HUVEC *in vitro* was monitored by using a commercially-available ELISA kit (BioSource International, Camarillo, CA) *and in vitro* bioactivity was studied by using an established MDCK cell serial dilution scatter assay [22].

### Transplantation

To prepare gels, HFH or Huh-7.5 cells and Bcl-2-HUVEC or a Bcl-2-HUVEC plus HGF-HUVEC mixture were suspended in rCI – human fibronectin (hFN) gels as previously described [12]. In some experiments, an additional 2.5 mg/ml murine liver-like basement membrane (mLBM; Cultrex, R&D Systems) originating from murine Engelbreth-Holm-Swarm (EHS) tumor and consisting of mouse collagen type IV, laminin, enactin and heparin sulfate proteoglycan, was added to the gel mixture. Each gel was prepared by using approximately 5×10^5^ HFH or Huh-7.5 and/or 1×10^6^ Bcl-2-HUVEC. The suspension was transferred to a 24-well plate (Becton Dickinson) and allowed to polymerize at 37°C. Following 18 hours in culture, polymerized gels were implanted in mice.

One flank of female SCID/bg mice was shaved by using clippers. Mice were anesthetized with 30% isoflurane in propylene glycol, disinfected with iodine followed by 70% ethanol, and then a 2 cm longitudinal incision was made in the dorsal flank and subcutaneous tissues bluntly dissected to create a subcutaneous pocket in the ventral abdominal wall. The gel was gently transferred to the pocket by using a spatula, taking care to place the gel under the subcutaneous fascia, and the incision closed with surgical staples. At the time of necropsy, gels were 1) fixed in 4% paraformaldehyde-PBS for 2–3 hours, then washed and transferred to saline, prior to paraffin-embedding for H&E (morphology) and immunohistochemical staining (see below) staining; or 2) snap frozen for qRT-PCR analyses as described below.

### Whole mount analysis

Immediately after tissue harvest, 2×2 mm unfixed gel samples were blocked by using PBS/1% BSA/5% normal mouse and rat serum on ice for 30 minutes. Fluorescently-labelled mouse anti-human and rat anti-mouse CD31 monoclonal antibodies (Immunotech and BD Biosciences, respectively) were added prior to incubation with occasional inversion on ice for 2–3 hours. After washing with PBS, samples were placed on slides and coverslipped as described [23].

### Immunohistochemistry (IHC)

Paraffin-embedded sections (5 µm) were de-paraffinized with xylene and then re-hydrated in ethanol and water. For antigen retrieval, sections were subjected to 95°C for 15 minutes in 10 mM sodium citrate buffer, pH = 6.0. Sections stained for Bcl-2 (DAKO), CK8/18 (Abcam), CK7 (Santa Cruz Biotechnologies, Santa Cruz, CA), and CD31 were also treated with 20 µg/ml proteinase K in 0.1 M Tris, pH 8.0, for 10 minutes at 37°C. Endogenous peroxidase activity was blocked with 0.3% hydrogen peroxide in methanol for 15 minutes. Slides were blocked in PBS-T/5% normal goat serum (blocking buffer for Bcl-2, CK8/18, and CD31) or PBS-T/5% BSA (blocking buffer for CK7) for 30 minutes at room temperature followed by avidin and biotin solutions (Vector Laboratories, Burlingame, CA) for 15 minutes each. Primary antibody (mouse anti-human Bcl-2, mouse anti-human CK8/18, mouse anti-human CD31, or goat anti-human CK7) diluted in blocking buffer was added and slides were incubated overnight at 4°C. After washing in PBS-T, bound antibody was detected with the appropriate biotin-labelled goat anti-mouse or donkey anti-goat IgG (H+L) (Jackson ImmunoResearch Laboratories), Vectastain ABC reagent system, and DAB substrate (Vector Laboratories). Finally, slides were counterstained with hematoxylin (Sigma).

### Transmission electron microscopy

To analyze cultured cells, primary HFH were grown on Primaria plasticware for 5 days prior to fixation with 2.5% glutaraldehyde in 0.1 M cacodylate buffer, pH 7.4, for 1 hour. Samples were postfixed for 1 hour in 1% osmium tetroxide in 0.1 M cacodylate buffer, stained in 2% uranyl acetate in 50 mM maleate buffer, and dehydrated in ethanol. Ultrathin sections (60 nm) were cut from epon blocks, stained with 2% uranyl acetate and lead citrate, and examined in a Tecnai 12 BioTWIN electron microscope (FEI/Phillips, Hillsboro, Oregon). To analyze gel tissue, 2×2 mm gel samples were fixed for 2 hours in freshly-prepared Karnovsky's buffer (2.5% glutaraldehyde/2% paraformaldehyde in 0.1 M cacodylate buffer) and analyzed as described above.

### Human protein ELISAs

Human albumin and human α-anti-trypsin levels in the mouse plasma was confirmed by using human-specific ELISA kits from Bethyl Laboratories, Montgomery, TX and Alpco Diagnostics Inc. Salem, NH, respectively, and human HGF levels in supernatants of *in vitro*-propagated HGF-HUVEC was determined by using a human-specific ELISA kit from R&D Systems.

### HCV

The cell culture-derived strain of HCV, Jc1, has been previously described [24], [25] and was propagated and titrated on Huh-7.5 [26]. The chimpanzee serum, “Rodney”, was a generous gift from Dr. Kris Krawczynski, Division of Viral Hepatitis, Centers for Disease Control and Prevention. The patient sera originated from a chronically-infected individual.

### Preparation of HCV-infected HFH/Bcl-2-HUVEC and Huh-7.5/Bcl-2-HUVEC gels

P0 HFH from a 16 week gestational age liver were expanded in T75 flasks for 4 days, as described above. HFH were washed once with HCM and either infected with HCV Jc1, “Rodney” or “Pat001” by using a 6 hour adsorption period with gentle rocking in HCM plus 2 mg/ml lipid rich albumin (LRA, Albumax I, Invitrogen) or mock-infected with the same media. Following the infection period, the inoculum was removed and flasks replenished with fresh HCM plus 2 mg/ml LRA. Twenty-four hours following, gels were prepared with HCV-infected and mock-infected gels and Bcl-2-HUVEC, as described above, and then implanted in mice. HFH were also grown in Primaria 6 well plates, infected as described above, and maintained *in vitro* for up to 8 days post-infection before detecting HCV by IHC, as described below.

### RNA extraction from gel tissue

At 2 weeks post-engraftment, mice were euthanized and gels retrieved. Tissue slices of 0.5×0.5×0.5 cm^3^ volume were immersed in 1 ml RNAlater (Ambion, Austin, TX) overnight at 4°C and then frozen at −80°C. To harvest RNA, the frozen block of RNAlater/gel was transferred to a foil pocket situated on a metal block that was pre-chilled by placing on dry ice and then the gel was retrieved by disrupting the block with a pestle. The frozen gel slice was transferred to a fresh, pre-chilled foil pocket, and the gel further disrupted into a powder by using a pestle. The powder was immediately dissolved in 600 µl RLT buffer, and incubated at 4°C for 15 minutes with agitation. Solution was homogenized by centrifuging through a QIAshredder spin column and then RNA extracted by using an RNeasy kit (both from Qiagen, Valencia, CA) according to the manufacturer's instructions. RNA was eluted from the column with 30 µl RNase-free water, aliquoted into 5 µl volumes and stored at −80°C until qRT-PCR analysis.

### qRT-PCR

RNA (100 ng/reaction) was amplified by using Brilliant II SybrGreen kit (Stratagene, La Jolla, CA) by using a 25 µl final volume in an Opticon cycler (MJ Research, Waltham, MA). The primers used in the current study are described in Table 1. Cycle conditions were as follows: 50°C for 30 minutes (RT) then 95°C for 10 minutes (RT denaturation), followed by 40 cycles of 95°C, 1 minute; 48°C, 1 minute; 60°C, 1 minute; and 72°C, 1 minute. Data were normalized by using GAPDH expression as internal controls, or in the case of HCV infections, expressed as threshold cycles (Ct).

**Table 1:** Primers used for qRT-PCR analyses.

	Forward Primer 5′ – 3′	Reverse Primer 5′ – 3′
GAPDH	GAAGGTGAAGGTCGGAGTC	GAAGATGGTGATGGGATTTC
albumin	CTTGCCTTGCTGAAAACACA	ACATTTGCTGCCCACTTTTC
HNF4α	CTGCTCGGAGCCACAAAGAGATCCATG	ATCATCTGCCACGTGATGCTCTGCA
CK7	TGAATGATGACATCAACTTCCTCAG	TGTCGGAGATCTGGGACTGC
HGF	TCAAAATAGATCCAGCACTG	GAAGTCCTTTATTCCTAGTAC
HCV	AAGACTGCTAGCCGAGTAGTGTT	GGTTGGTGTTACGTTTGGTTT

### HCV immunocytochemistry and immunoelectron microscopy

HCV Jc1- and mock-infected HFH cultures were prepared as described above, and then maintained *in vitro* for various times post-infection. HCV replication was determined by staining for the HCV non-structural (NS) protein NS5A, as previously described [26]. Briefly, cells were fixed in methanol at −20°C for 10 minutes. Following 2 washes with PBS, endogenous peroxidases were quenched with 3% hydrogen peroxide in methanol for 15 minutes at room temperature. Cells were blocked for 30 minutes at room temperature with blocking buffer consisting of PBS/T containing 1% BSA and 0.2% dried skim milk. Cells were incubated with anti-NS5A mouse monoclonal antibody (clone 9E10 [27], provided by Dr. Charles Rice, final concentration of 40 ng/ml,) for 60 minutes at room temperature. Following 2 washes with PBS, horse radish peroxidase-labelled anti-mouse IgG (ImmPress, Vector Laboratories) was allowed to react for 30 minutes at room temperature. Following 2 additional washes with PBS and 1 wash with PBS-T, DAB (DAKO) was added and color allowed to develop for 1–10 minutes. Cells were counter-stained with hematoxylin.

HCV replication was detected in tissue sections by staining for the HCV NS5A by IEM. Briefly, ultrathin 70 nm sections were cut on a Leica FC6 Cryoultramicrotome and collected on formvar/carbon coated grids as previously described [28]. Grids were placed section side down on drops of 0.1 M ammonium chloride for 10 minutes to quench untreated aldehyde groups, then blocked for non-specific binding in 1% fish skin gelatin (FSG) in PBS for 10 minutes. Following extensive washing in PBS, grids were incubated with mouse monoclonal antibody against HCV NS5A (clone 9E10, 1∶500 dilution in 1% FSG/PBS) for 30 minutes. After additional washes, grids were incubated with a bridging rabbit anti-mouse serum (Jackson ImmunoResearch Laboratories, Inc; 1∶200 in 1% FSG/PBS) for 30 minutes, rinsed and incubated on protein A gold-10 nm beads (University Medical Center, Utrecht, Netherlands; 1∶75 dilution in 1% FSG/PBS). Grids were washed, fixed by using 1% glutaraldehyde/PBS for 5 minutes, rinsed again, transferred to a methyl cellulose/0.5% uranyl acetate mixture for 10 minutes, and then dried. Samples were viewed at 80 Kv with a FEI Tecnai Biotwin election microscope and images recorded by using a Morada CCD and iTEM (Olympus, Center Valley, PA) software.

## Results

### *In vitro* characterization of cultured HFH

**Figure 1:**
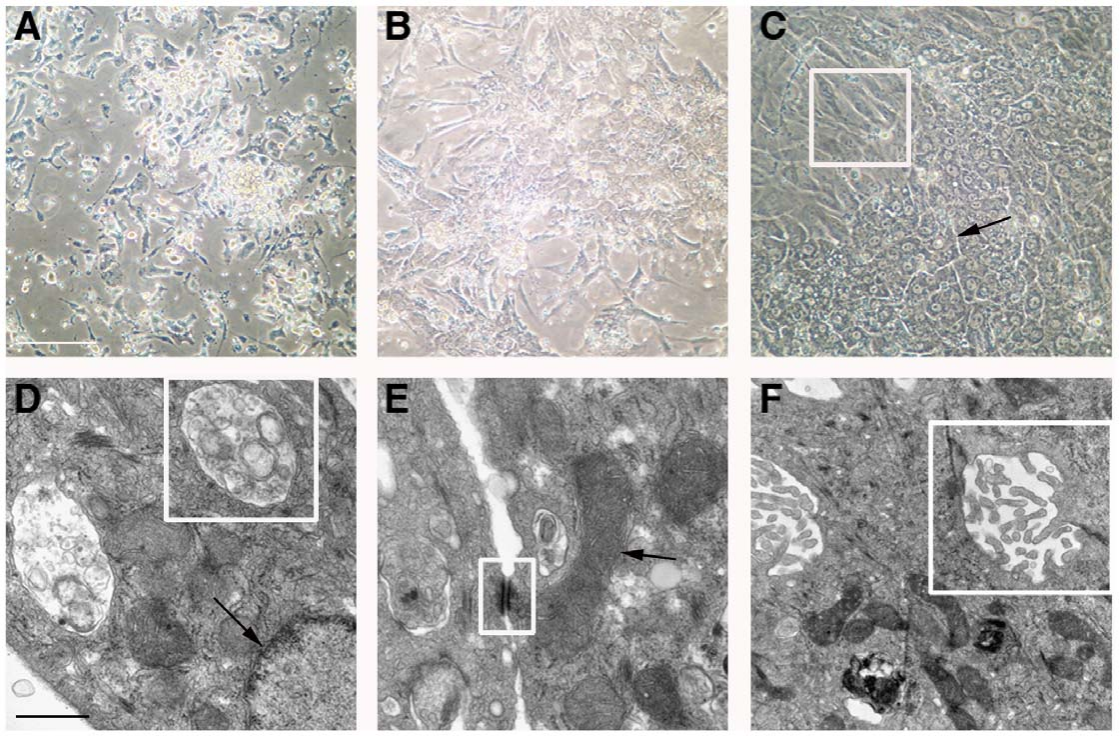
Morphologic analysis of cultured HFH. Phase contrast micrographs (A–C) show primary cell cultures on days 1 (A), 3 (B), and 5 (C) following initial harvest. Panel C also demonstrates the heterogeneity of the cultured cell population, consisting of large regions of polygonal epithelial cells with adjacent bile canaliculi (black arrows) and spindle-shaped intervening stromal cells (box region). Electron micrographs (D–F) of day 7 cultures confirm the presence of HFH with normal ultrastructural features. D: multivesicular bodies and nuclei (box and arrow, respectively); E: desmosomes and mitochondria (box and arrow, respectively); F: canalicular villi and tight junctions (box). Size bars: 100 µm (A–C); 1 µm (D–F).

**Figure 2:**
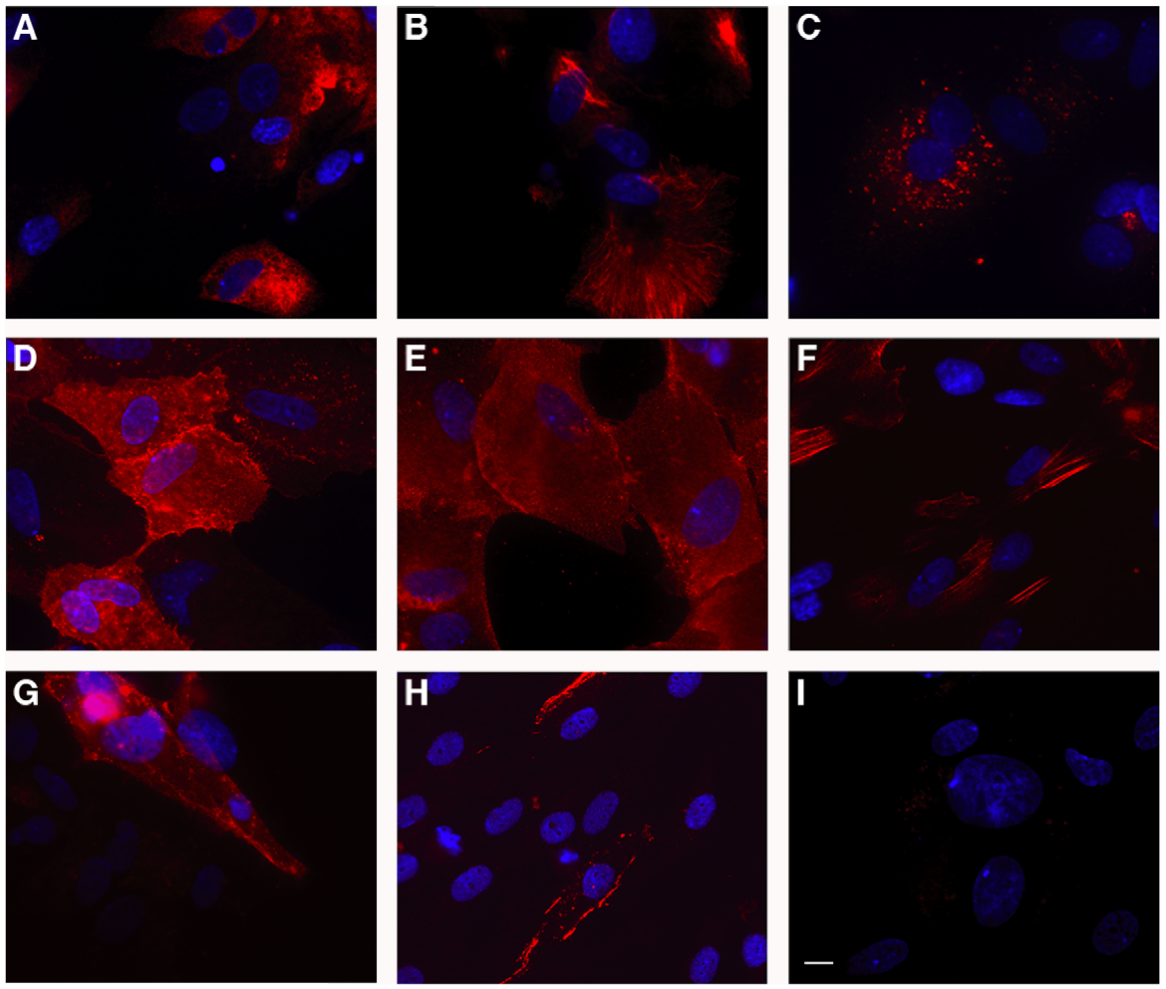
Immunofluorescence staining analysis of HFH. HFH were harvested from 15 week gestational age HFL tissue and cultured on collagen type I-coated coverslips for 8 days in chemically-defined HCM. Cells were fixed with 4% paraformaldehyde and permeabilized and blocked with either PBS-T plus 1% gelatin (albumin detection) or PBS-T plus 1% BSA (for all other intracellular or cell surface markers). DAPI was also added to identify cell nuclei. The cultures were stained for albumin (A), CK8/18 (B), HepPar1 (C), HEA125 (D), CK19 (E), SMA (F), CD31 (G), or VE-cadherin (H). Unconjugated primary antibodies (all except CD31-PE and VE-cadherin-PE) were detected with a Cy3-labelled anti-mouse secondary antibody. HFH stained with secondary antibody alone served as a negative control (I). Photos were captured at a 63x magnification on a Zeiss fluorescence microscope using OpenLab Imaging Software (Improvision, Waltham, MA). Size bar indicates 15 µm.

Cells harvested from 15-16 week gestational age human fetuses were attached to Primaria tissue culture plates in the presence of 5% FCS for 4–6 hours, then cultured in chemically-defined medium (HCM) in the absence of serum. The cells initially attached as clumps (Fig. 1A) but over the next 3–5 days (Fig. 1B and 1C) spread to form regions of polygonal cells with round nuclei and prominent nucleoli, surrounded by a monolayer of stromal cells (Fig. 1C, box). Relatively wide intercellular spaces (Fig. 1C, arrows) resembling epithelial canalicular surfaces were frequently observed. Examination by transmission electron microscopy (TEM) revealed features consistent with healthy and functionally active hepatic epithelial cells in day 7 primary cultures. Specifically, the presence of normal multivesicular bodies and nuclei (Fig. 1D, box and arrow, respectively), desmosomes and mitochondria (Fig.1E, box and arrow, respectively), and canalicular villi and tight junctions (Fig. 1F, box) indicated the presence of healthy hepatocytes through at least 1 week of culture when maintained on cationic plates in HCM. The polygonal cells present in cell clumps were likely bipotential HFH—which normally develop into either mature hepatocytes or cholangiocytes *in vivo*—as judged by their consistent co-expression of 1) albumin and HepPar1 (Fig. 2A and 2C), expressed in hepatocytes and their progenitors, 2) HEA125 and CK19 (Fig. 2D and 2E), expressed in cholangiocytes and their progenitors, and 3) CK8/18, expressed in both cell types (Fig. 2B). Some elongated cells expressed the stellate cell or the vascular smooth muscle cell marker SMA (Fig. 2F). A few spindle-shaped cells expressed the endothelial cell (EC) marker CD31, and yet even fewer cells of the same morphology expressed VE-cadherin (Fig. 2G and 2H, respectively). Cells stained with secondary antibody alone are shown in Fig. 2I, as a negative control. The HFH cultures could be passaged twice with EGTA/collagenase treatment; however, at passage 3 or later, the proportion of polygonal epithelial-appearing cells declined, presumably due to overgrowth by stromal cells. Passage 0 and 1 HFH cultures consistently produced high levels of albumin (i.e. >300 ng/ml media/24 hours).

### Morphologic features of gels containing HFH following *in vivo* engraftment

**Figure 3:**
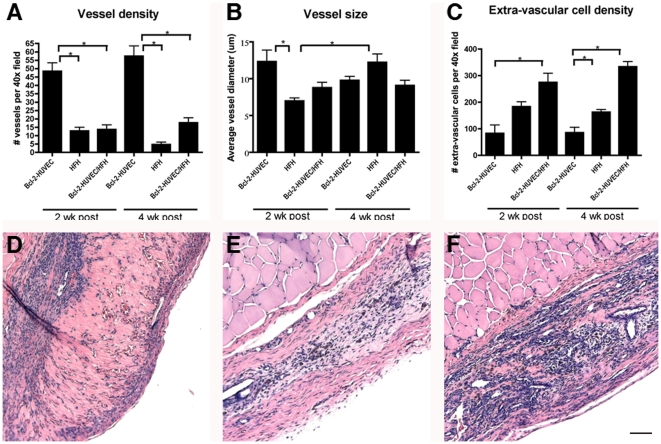
Quantitative analysis of vessel and extra-vascular cell numbers in gels incorporating Bcl-2-HUVEC and/or HFH. Panels A-C show vessel density and size (A and B, respectively) and number of extra-vascular cells (C) calculated from H&E-stained sections of vascularized gels harvested at 2 and 4 weeks post-engraftment. Results are expressed as the mean ± standard error of the mean (SEM) and asterisks indicate significance at p≤0.05. Panels D–F show representative H&E-stained micrographs from gels harvested at 4 weeks post-engraftment containing Bcl-2-HUVEC (D), HFH (E), and HFH/Bcl-2-HUVEC (F). Size bar: 100 µm.

Simple rCI-hFN gels containing HFH cultures only (i.e. without Bcl-2-HUVEC) were prepared and then incubated for 18 hours *in vitro* prior to transfer to a subcutaneous pocket in SCID/bg mice. At both 2 and 4 weeks following transplantation, all gels were 4×3×2 mm^3^ in size (data not shown). Three of 4 and 2 of 3 HFH gels appeared to be vascularized based on hematoxylin and eosin (H&E)-stained tissue sections at 2 and 4 weeks post-engraftment, respectively. Specifically, vascularized gels contained an average of 12.9 and 4.7 recognizable vessels per 40× field at 2 and 4 weeks post-engraftment, respectively (Fig. 3A). Though vessels of capillary size (<10 µm) were found in vascularized HFH grafts at both 2 and 4 weeks post-engraftment, vessels were significantly larger (p<0.05; with the largest vessel being 22.2 µm) at the latter time point (Fig. 3B). Nevertheless, the grafts did not appear to be highly perfused at either time point as judged by general pallor.

Because CD31+ EC were observed in the HFH cultures prior to gel formation and implantation (Fig. 2G), we analyzed whether vessels in HFH grafts were donor (human)- or host (mouse)-derived. *In vitro* analysis of pre-implantation HFH gels confirmed the presence of tubes (Fig. S1A, arrows), possibly indicative of early vessel formation and a potential donor contribution to the observed microvascular network. Human- and mouse-specific CD31 immunostaining of unfixed HFH gels at harvest confirmed that vessels were largely of donor origin: an elaborate trabecular network of human CD31-lined tubes comprised the bulk of the gel (Fig. S1B) while larger vessels positive for mouse CD31 were infrequently observed and confined to the gel periphery (Fig. S1C; arrow demarcates gel edge). Capillaries present in HFH gels at 2 weeks post-engraftment (Fig. S1D, arrows) were lined with human CD31-expressing cells based on IHC staining (Fig. S1E). Even though vascularized HFH grafts appeared poorly perfused based on a lack of red blood cells (RBC) in H&E-stained sections at 4 weeks post-engraftment, some RBC-filled capillaries were identified by TEM analysis (Fig. S1F, arrows).

**Figure 4:**
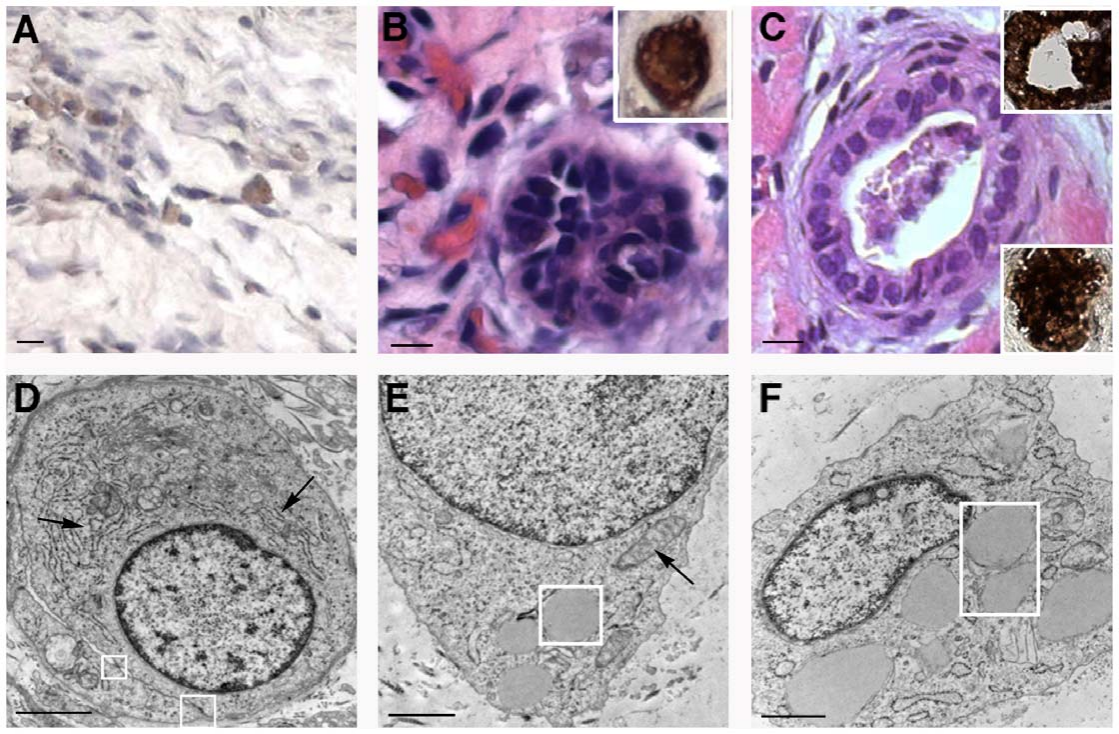
Morphology of gels containing passage 1 HFH implanted at an ectopic site. A: H&E-stained section from a graft harvested at 2 weeks post-engraftment showed the presence of RBC-filled capillaries and a duct-like structure lined with columnar epithelial cells that stain intensely positive for CK8/18 (inset). B. At 4 weeks post-engraftment, a small proportion of the unorganized extra-vascular cells within the HFH gel stroma stained positive for CK8/18. Staining was absent in similarly-stained Bcl-2-HUVEC alone gels. C: H&E-stained sections from a graft harvested at 4 weeks post-engraftment showed a duct with a lumen that stains intensely for CK8/18 (top inset) and CK7 (bottom inset). D, E: TEM analyses identified metabolically active HFH possessing extensive rough endoplasmic reticulum (D, arrows), tight junctions (D, box) with an immature intervening canalicular region, phenotypically normal mitochondria (E, arrow), and lipid droplets (E, box). F: Quiescent stellate cells harboring numerous large fat droplets (box) were also observed. Size bars: 10 µm (A-C), 2 µm (D, E), and 1 µm (F).

Implant cellularity appeared to correlate with the degree of vascularity. In more vascularized gels, a proportion of the extra-vascular cells were identified as unorganized hepatic epithelial cells based on their expression of CK8/18 by IHC (Fig. 4A). Specifically, the average number of CK8/18+ cells at 2 weeks post-engraftment was 10.8 +/− 3.6 per high-power field. Some hepatic epithelial cells staining intensely positive for CK8/18 self-organized, forming putative bile ducts (Fig. 4B and 4C, micrograph and top IHC insets) that occasionally possessed a lumen (Fig. 4C). These structures also stained positive for CK7, a marker expressed by cholangiocytes but not hepatoblasts or hepatocytes (Fig. 4C, bottom inset). TEM analysis revealed hepatoblast-like epithelial cells (Fig. 4D and 4E) containing prominent round nuclei and cytosol rich in rough endoplasmic reticulum (Fig. 4D, arrows), mitochondria (Fig. 4E, arrow), and occasional lipid droplets (Fig. 4E, box). Although “canalicular” regions limited by tight junctions were observed (Fig. 4D, box), the lack of microvilli within the non-expanded “canalicular” space suggested an immature phenotype. Quiescent stellate cells, characterized by their star-shaped morphology and large intracellular lipid droplets (Fig. 4F, box), were also noted by TEM.

### Morphologic features of gels containing HFH/Bcl-2-HUVEC following *in vivo* engraftment

Previous studies have shown that a mature microvasculature network develops and is maintained for at least 2 months following engraftment of Bcl-2-HUVEC in rCI-hFN gels when placed within the abdominal wall of SCID/bg mice [12], [17]. We therefore tested whether engraftment and viability of HFH at ectopic sites would increase when co-transplanted with Bcl-2-HUVEC. Vessel density was significantly lower in the HFH and HFH/Bcl-2-HUVEC gels at both 2 and 4 weeks post-engraftment compared to grafts containing Bcl-2-HUVEC alone (Fig. 3A; p<0.001). In other words, co-engraftment with HFH appeared to inhibit vessel formation by Bcl-2-HUVEC. At the same time, compared to gels containing Bcl-2-HUVEC or HFH alone, co-transplants of Bcl-2-HUVEC and HFH in simple rCI-hFN gels were consistently larger (6×4×3 mm^3^) and, compared to HFH alone, appeared much more well-perfused by gross observation (Fig. S2A). Vessel diameters were significantly smaller in HFH implants compared to Bcl-2-HUVEC gels at 2 weeks post-engraftment only (7.0 +/− 0.4 versus 12.3 +/− 1.6 µm; Fig. 3B, p<0.01). Although the average vessel size did not significantly differ between the Bcl-2-HUVEC and the HFH/Bcl-2-HUVEC co-engrafted gels at 4 weeks post-engraftment (Fig. 3B; 9.8 µm versus 9.1 µm), multi-layered vessels were more frequently observed in the HFH/Bcl-2-HUVEC co-seeded gels (Fig. S2B) compared to the Bcl-2-HUVEC implants (Fig. S2C) at both time points. Staining for human and mouse CD31 revealed a developed human microvascular network in the center of the gel (Fig. S2D and S2E) surrounded by mature, investing murine vessels at the gel edge (Fig. S2F). Based on IHC, most small caliber vessels were lined with human CD31-expressing EC, whereas larger vessels contained mouse CD31+ EC or a combination of human CD31+ and mouse CD31+ EC (data not shown). TEM analyses did not reveal intercellular or intracellular fenestrations between EC, typical of sinusoidal endothelium, in either HFH or HFH/Bcl-2-HUVEC grafts.

**Figure 5:**
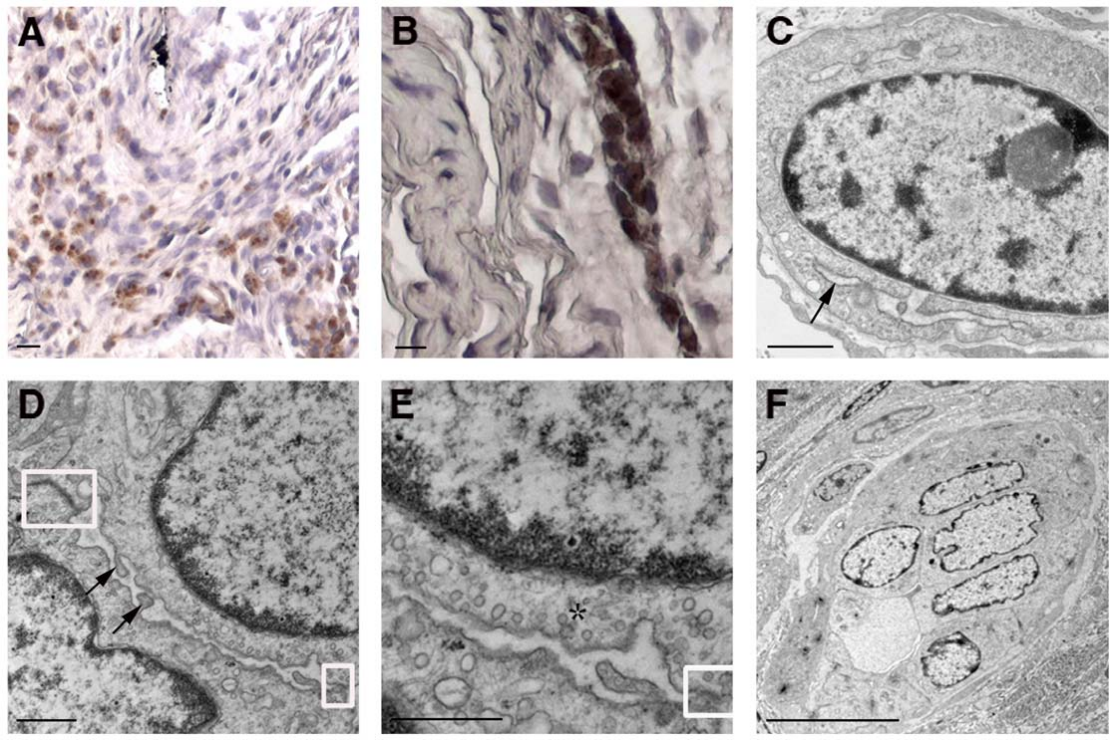
Morphology of gels containing passage 1 HFH/Bcl-2-HUVEC implanted at an ectopic site. Many extra-vascular cells in the HFH/Bcl-2-HUVEC gels harvested at 2 weeks post-engraftment stained positive for CK8/18 (A) and appeared more organized by 4 weeks post-engraftment (B). Immature HFH, characterized by their oval cell and nuclear shape, few mitochondria, and wide, undeveloped Golgi (C, arrow) were confirmed by TEM. More mature hepatic epithelial cells (D, E; Hep) possessed characteristic large circular nuclei and bile canaliculi (box) with apical microvilli (arrows). Areas of clathrin-coated pits and coated vesicles indicated endocytic activity at the canalicular membrane surface (E, asterisk). Columnar epithelial cells were shown to organize into rudimentary ductules (F). Size bars: 100 µm (A), 10 µm (B, F), 1 µm (C, D), and 0.5 µm (E).

At 4 weeks post-engraftment, the total number of extra-vascular cells within the gel stroma was significantly higher in the HFH/Bcl-2-HUVEC gels (333.9 +/− 18.0) compared to the additive numbers of extra-vascular cells in the Bcl-2-HUVEC gels (85.6 +/− 11.4) and HFH gels (162.7 +/− 14.4; see Fig. 3C). This same effect was not observed at 2 weeks, suggesting that increased vessel numbers promoted HFH expansion and survival. Consistent with our original hypothesis that inadequate perfusion limits the growth and survival of non-transformed heptocytes following implantation, significantly greater numbers of hepatic epithelial cells were observed in HFH/Bcl-2-HUVEC grafts compared to HFH grafts based on CK8/18 staining (Fig. 5A). Specifically, an average of 37.2 and 10.8 CK8/18+ cells were observed per 40× field at 2 weeks post-engraftment, representing 13.5% and 5.9% of the extra-vascular cell pool in HFH/Bcl-2-HUVEC gels and HFH gels, respectively. At 4 weeks post-engraftment, some of the CK8/18+ cells appeared to associate in structures resembling parenchymal cords (Fig. 5B). The presence of HFH and/or more mature hepatocytes in HFH/Bcl-2-HUVEC grafts was confirmed by TEM analyses (Fig. 5C-5E). Immature HFH were identified based on the oval shape of the nucleus (Fig. 5C) and wide, under-developed Golgi cisternae (Fig. 5C, arrow). Maturing, active hepatocytes were identified by round nuclear morphology (Fig. 5D and 5E), presence of tight junctions between adjacent cells, apical canalicular microvilli (Fig. 5D, boxed regions and arrows, respectively), and characteristic apical membrane region containing coated pits and pericanalicular vesicles (Fig. 5E, asterisk). Epithelial cells were also noted to organize into rudimentary ductules by TEM in HFH/Bcl-2-HUVEC grafts (Fig. 5F). Analysis of representative Bcl-2-HUVEC, HFH, and HFH/Bcl-2-HUVEC gels confirmed a cell viability of >97% based on TUNEL assay (data not shown).

### HFH function following engraftment in gels in immunodeficient mice

**Figure 6:**
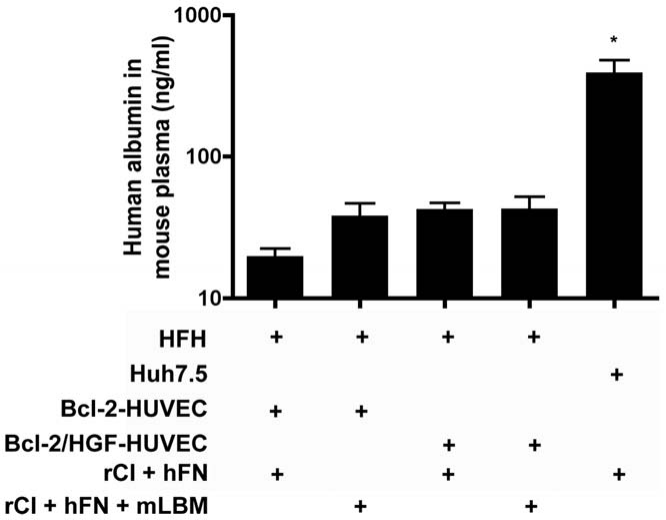
Quantitation of human albumin in the plasma of engrafted mice. Human albumin (20 ng/ml) was detected in the plasma of mice co-engrafted 2 weeks earlier with HFH and Bcl-2-HUVEC suspended in rat tail collagen I (rCI) and human fibronectin (hFN) (left bar). The albumin concentration increased to 40 ng/ml when gels also contained murine liver-like basement membrane (mLBM) proteins or HGF-transduced HUVEC, however no further increases in albumin secretion were observed by using a combination of mLBM plus HGF-HUVEC (middle bars). Highest levels of albumin (average of 392 ng/ml) were observed in mice harboring Huh-7.5/Bcl-2-HUVEC gels (right bar). Data is expressed as ng/ml human albumin in mouse plasma +/− S.E.M.

Passage 0 and 1 HFH cultures consistently produced high levels of albumin prior to engraftment (i.e. >300 ng/ml media/24 hours) and low levels of human albumin were present in the plasma of immunodeficient mice harboring grafts of HFH/Bcl-2-HUVEC. Specifically, an average of 19.6 ng/ml albumin was detected in the plasma of mice engrafted with our base model of HFH/Bcl-2-HUVEC suspended within a rCI/hFN protein gel (Fig. 6, left bar). However, mice engrafted with human hepatoma cells (Huh-7.5) within an identical gel system continued to produce high human albumin levels (390 ng/ml plasma; Fig. 6, right bar, p<0.001). Therefore, it appeared that while the non-transformed, bipotential HFH were surviving, a large proportion might be undergoing de-differentiation and/or progression towards a differentiated cholangiocyte upon engraftment, based on plasma albumin levels.

### Strategies to promote and preserve HFH function

**Figure 7:**
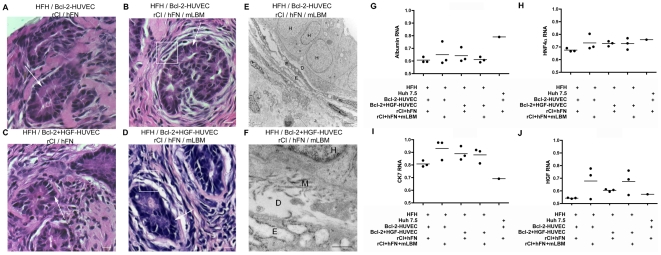
Effect of mLBM and/or HGF-HUVEC on morphology and gene expression in vascularized gels containing HFH. A–D: At 2 weeks post-engraftment, large clusters of HFH (white arrows) were observed in gels containing HFH, Bcl-2-HUVEC and rCI/hFN alone (A), or with additional mLBM (B), additional HGF-HUVEC (C), or additional mLBM and HGF-HUVEC (D). HFH clusters were surrounded by multiple layers of endothelial cells when cast in gels containing mLBM (white boxes in B and D). E–F: TEM analyses confirmed the presence of a Disse-like space *in vivo*, bordered by HFH and endothelial cells and containing matrix proteins (letter D, H, and E, respectively, in panels E and F and letter M in panel F). Size bars: 10 µm (A–D), 2 µm (E), and 500 nm (F). G–I: Analysis of mRNA expression in gel tissue revealed loss of hepatocyte phenotype in HFH-containing gels, based on lower levels of albumin (G) and HNF4α (H), and higher levels of CK7 (I), compared to Huh-7.5-containing gels. Albumin and HNFα expression (G and H, respectively) was slightly better maintained by inclusion of LBM and/or HGF-HUVEC. The presence of continued HGF expression *in vivo* was confirmed by HGF qRT-PCR (J) and most prominent in gels containing mLBM. Data in panels G–I is expressed as a ratio of target gene mRNA to GAPDH mRNA internal control.

We therefore tested additional approaches designed to maintain more HFH in a differentiated state within vascularized gels: 1) both Bcl-2-HUVEC and HGF-HUVEC were included within the gel; 2) additional mLBM, including mouse laminin, collagen type IV and heparin sulfate proteoglycans were added to the protein gel; and 3) both strategies were combined. Prior to engraftment, we confirmed that HGF-transduced HUVEC secreted 10 ng/ml HGF into the supernatant and exhibited appropriate bioactivity in an MDCK cell-based scatter assay (Fig. S3). Slightly higher (but statistically insignificant) increases in human albumin (i.e. to 40 ng/ml) were detected in the plasma of mice engrafted with Bcl-2- and HGF-HUVEC or in gels containing mLBM (Fig. 6, middle bars). No additional increases were noted if both conditions were included within the same protein gel. Histological analyses revealed that HFH appeared as clusters of columnar epithelial cells that were organizing into duct-like structures in gels prepared by using all 4 protocols: Bcl-2-HUVEC and rCI/hFN, Bcl-2-HUVEC and rCI/hFN/mLBM, Bcl-2+HGF-HUVEC and rCI/hFN, and Bcl-2+HGF-HUVEC and rCI/hFN/mLBM (Fig. 7A–7D, respectively, white arrows) by 2 weeks post-engraftment. Intriguingly, one structural difference was noted in the HFH/EC interactions within the various treatment groups. Specifically, in gels containing mLBM, the HFH clusters were surrounded with multiple layers of EC (white box in Fig. 7B and 7D). TEM analyses confirmed the presence of a Disse-like space containing matrix proteins between the HFH and EC (Fig. 7E and 7F, box). However, the underlying EC did not appear to contain fenestrations characteristic of true hepatic sinusoids. We then analyzed expression of several liver-specific genes within the gel implant, specifically human albumin (expressed by both HFH and more mature hepatocytes), huma hepatic nuclear factor 4α (HNH4α; expressed in differentiated hepatocytes) and human CK7 (found in mature cholangiocytes). The expression of albumin mRNA was slightly higher in the Bcl-2+HGF-HUVEC and mLBM groups compared to Bcl-2-HUVEC/rCI/hFN group (Fig. 7G, GAPDH ratio of 0.65 versus 0.60), whereas HNF4α expression was higher in all 3 treatment groups (Fig. 7H, GAPDH ratio of >0.73 in Bcl-2-HUVEC and rCI/hFN/mLBM, Bcl-2+HGF-HUVEC and rCI/hFN, and Bcl-2+HGF-HUVEC and rCI/hFN/mLBM groups) compared to the base Bcl-2-HUVEC and rCI/hFN group (GAPDH ratio of 0.66). Intriguingly, even though levels of albumin expression were lower in all types of HFH gels compared to the Huh 7.5 gel (Fig. 7G), HNH4α expression in the HFH gels containing additional mLBM, HGF-HUVEC or both components (Fig. 7H) approached levels found in the Huh-7.5 gel, perhaps indicating that some HFH were maintained in a differentiated state. However, CK7 was also mildly increased (ie. Fig. 7I, GAPDH ratio of >0.88 in Bcl-2-HUVEC and rCI/hFN/mLBM, Bcl-2+HGF-HUVEC and rCI/hFN, and Bcl-2+HGF-HUVEC and rCI/hFN/mLBM groups versus a ratio of 0.8 in Bcl-2-HUVEC and rCI/hFN group). Therefore, it appeared that some cells were differentiating towards a cholangiocyte phenotype. None of the aforementioned changes in HFH gene expression amongst the various treatment groups were pronounced. Huh-7.5 cells continued to express high levels of albumin and HNF4α and low levels of CK7 *in vivo* (Fig. 7G-7I). HGF expression was highest in gels containing additional mLBM (Fig. 7J).

### Replication of HCV Jc1 infection in HFH in HFH/Bcl-2-HUVEC gels *in vivo*

**Figure 8:**
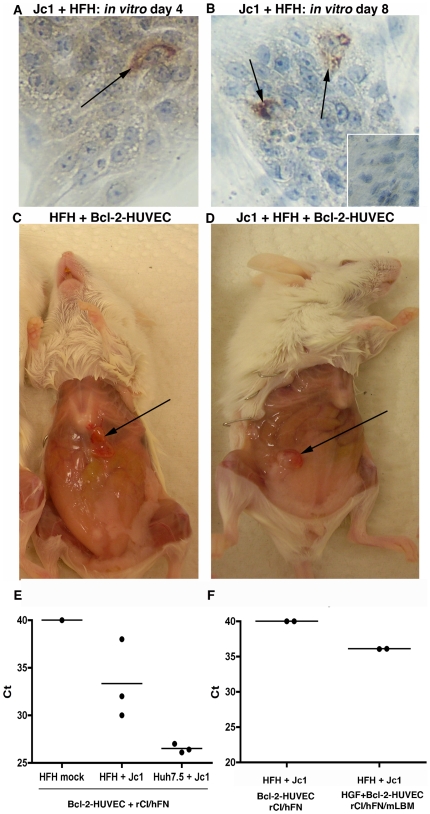
*In vitro* and *in vivo* maintenance of HCV Jc1 infection in HFH. A, B: IHC staining for HCV NS5A revealed rare positive HFH at day 4 and day 8 post-infection (arrows in A and B, respectively). Similarly stained mock-infected HFH cultures confirmed the specificity of the HCV NS5A IHC assay (B, inset). C, D: Mock- or HCV Jc1-infected HFH cells were co-engrafted with Bcl-2-HUVEC and a rCI/hFN matrix on the abdominal wall of SCID/bg mice (C and D, respectively). Representative gels harvested at 2 weeks post-engraftment are highlighted by black arrows. E, F: qRT-PCR analysis revealed the presence of HCV RNA in Jc1-infected HFH gels (E, middle column), but not in mock-infected gels (E, left column). Analysis of mRNA extracted from HCV Jc1-infected Huh-7.5-containing gel tissue was used as a positive control (E, right column). Increased levels of HCV Jc1 RNA were detected in gels containing additional HGF-HUVEC and mLBM, compared to the Bcl-2-HUVEC and rCI/hFN gel system (F, right versus left column, respectively). Data points represent individual gels.

**Figure 9:**
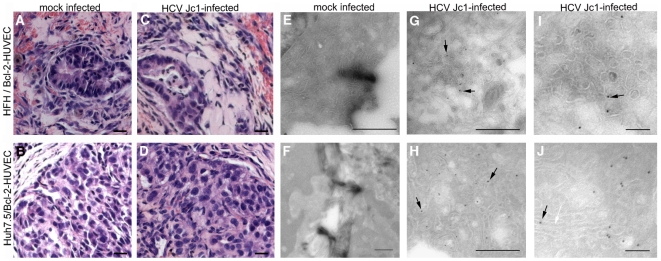
Histological and IEM analysis of HCV Jc1-infected HFH and Huh-7.5. Large numbers of epithelial-appearing cells were observed in Bcl-2-HUVEC gels harvested at 2 weeks post-engraftment and containing either mock-infected HFH or Huh-7.5 cells (A and B, respectively) or HCV Jc1-infected HFH or Huh-7.5 (C and D, respectively). Staining for HCV NS5A by IEM revealed minimal background stain in both mock-infected HFH and Huh-7.5 (E and F, respectively), in contrast to the presence of HCV NS5A protein immunogold stain associated with ER membranes observed in infected HFH and Huh-7.5 gels (G and H, respectively, black arrows). Panels I and J are higher magnifications of infected HFH and Huh-7.5 cells within the gels. White arrow in J indicates ER membrane. Size bars: 20 µm (A–D), 1 µm (E, F), 500 nm (G, H), and 100 nm (I, J).

Following confirmation of long-term survival of HFH within Bcl-2-HUVEC gels, we next studied whether engrafted HFH would support infection of the tissue tropic HCV Jc1 isolate *in vivo*. In a preliminary experiment, we confirmed that low percentages of HFH indeed sustained a HCV Jc1 infection *in vitro*. At a multiplicity of infection of 1, *in vitro* HFH cultures consistently become infected with HCV Jc1, based on HCV NS5A+ cells. Specifically, immunostaining for HCV NS5A revealed that <1 in 10,000 cells supported HCV Jc1 replication at days 4 and 8 post-infection (Fig. 8A and 8B, respectively; representative positive-stained HFH shown at the black arrows). In contrast, neither NS5A+ cells nor HCV RNA in the culture medium were observed in HFH cultures adsorbed with serum originating from a chronically-infected human or chimpanzee (data not shown). Next, total RNA from gel tissue containing non-infected and Jc1-infected HFH (representatives highlighted with black arrows in Fig. 8C and 8D, respectively) was extracted and subjected to HCV qRT-PCR. HCV RNA was detected in both HFH/Bcl-2-HUVEC (n = 2/3) and Huh-7.5/Bcl-2-HUVEC (n = 3/3) gel tissue, but not from RNA extracts originating from a non-infected gel (Fig. 8E), nor in gels infected ex vivo with serum from a chronically infected chimpanzee or human patient (data not shown). In a follow-up infection experiment, HCV RNA was not detected in infected HFH/Bcl-2-HUVEC (n = 0/2) cast in rCI/hFN proteins, however was present in a matched gel prepared with additional HGF-HUVEC and mLBM (Fig. 8F, n = 2/2). Epithelial cells were maintained in both non-infected and infected HFH/Bcl-2-HUVEC and Huh-7.5/Bcl-2-HUVEC grafts (Fig. 9A–9D) for the duration of the *in vivo* culture period based on H&E staining. Furthermore, no phenotypic changes were apparent between the mock- and virus-infected HFH or Huh-7.5 gels, based on gross or histological observations (Fig. 9A versus 9C and Fig. 9B versus 9D). The presence of HCV replication was determined by using IEM methods specific for HCV NS5A. Immunogold background stain was only rarely observed in non-infected HFH and Huh-7.5 gel tissue (Fig. 9E and 9F). In contrast, immunogold staining was observed in both HFH and Huh-7.5 (Fig. 9G–9J, black arrows), confirming that both epithelial cell types supported HCV Jc1 replication *in vivo*. Similar to our qRT-PCR analyses, the number of infected cells and amount of NS5A per cell was higher in the Huh-7.5 gel, compared to the HFH gel (Fig. 9H and 9G, respectively). NS5A staining was typically associated with ER membranes (Fig. 9J, white arrow).

## Discussion

We report here on the development of a simple model of primary human hepatoblast and hepatoma cell engraftment and HCV infection in healthy, adult immunodeficient mice. We chose to use HFH, rather than human adult hepatocytes, for several reasons: 1) these cells are routinely available thus well-suited for scheduled transplantation experiments; 2) HFH may be expanded *in vitro* by using conditions that maintain typical hepatocytic morphology and gene expression patterns even after several months of culture [15]; 3) HFH may be transduced with lentiviruses *in vitro* [29], [30]; and 4) HFH may be combined with hematopoietic stem cell (HSC) co-engraftment from the same human fetal liver [13], [31] to investigate the development of an immunocompetent mouse model for studies of HCV pathogenesis and pre-clinical testing of prophylactic and therapeutic HCV vaccines. Despite encouraging successes of high levels of rodent hepatoblast expansion within the liver parenchyma of immunodeficient mice [32]–[34], similar degrees of liver reconstitution have not been achieved following transplantation of early human liver progenitors [29], [35]–[39]. Therefore, it appears that the murine liver environment lacks the appropriate signals for HFH maturation and/or expansion within the liver parenchyma. The use of subcutaneous implants of HFH or even earlier progenitors, such as hepatocyte-like cells derived from human embryonic stem cells [40], [41], circumvents the endogenous murine liver environment and allows for the rational design of a matrix that can sustain large number of HFH that are infectable with HCV.

One possible reason for the limited prior successes of the heterotopic approach for hepatic epithelial cell transplantation is that cell survival is hampered by the lack of rapid neovascularization to support graft perfusion [42]–[44]. A delay in thorough vascularization of the implant area creates an environment low in oxygen and other nutrients and likely contributes to the initial death of transplanted cells [45]. In general, cells positioned more than 200 µm from a blood vessel suffer from hypoxia and die [46], and cell death is especially significant for cells with high metabolic activity, such as hepatocytes [42]. Sustained-release microspheres containing angiogenic factors promote enhanced short-term (up to 2 weeks) vascularization and rodent hepatocyte viability [47]–[52]. We report here that the vasculature present in the HFH/Bcl-2-HUVEC gels supported higher numbers of CK8/18+ hepatic epithelial cells compared to HFH gels, likely through the provision of oxygen and other nutrients delivered by blood perfusion. Alternatively, direct EC-hepatocyte contact or paracrine signaling may have facilitated hepatocyte survival and development [53]–[55].

We noted that the inclusion of either mLBM or HGF-transduced HUVEC supported increased production of human albumin by engrafted HFH and as well sustained higher levels of HCV Jc1 replication. Systemic administration of HGF in normal and liver-injured mice [56], [57] and rats [58] caused a hepatotrophic effect that altered liver-associated serum biochemistries. A deletion variant of HGF, lacking 5 consecutive amino acids from the kringle domain, results in higher mitogenic activity in rat hepatocytes compared to full-length HGF [59] and provides additional protective effects for hepatocyte transplantation in liver injury models [60]. A further benefit of systemic delivery of HGF in our studies stems from the fact that HGF also enhances VEGF-induced angiogenesis [61] and prevents EC apoptosis [62]. Liver matrix proteins, produced mainly by resident hepatic stellate cells [63], [64], provide structural and behavioral support for epithelial cells and EC. Liver matrix proteins consist of typical basement membrane components (laminins 1–4, nidogen, enactin, heparin sulfate proteoglycans and type IV collagen), and also includes the non-basement membrane constituents type I collagen and fibronectin [65], [66]. Interactions between the hepatocyte and its unique basement membrane are important for maintaining hepatocyte survival and differentiated function [66], [67]. In fact, changes in the liver protein matrix, such as the loss of the α4 and α6 isoforms of type IV collagen [68], are hypothesized to contribute to decreased hepatocyte viability during the progression of liver cirrhosis [66], [69], [70]. A laminin-rich matrix has recently been shown to promote differentiation of mouse fetal hepatoblasts both *in vitro* and *in vivo* [71], supporting our findings of higher levels of albumin production when HFH are cast in gels with a mLBM. Furthermore, it is possible that co-engrafted human stellate cells produce a typical liver matrix within the gel stroma that may help to support hepatocyte survival in addition to the normoxic environment provided by Bcl-2-HUVEC and fetal liver-derived EC [51].

The levels of HCV RNA detected in the gel parenchyma were higher in Huh-7.5-containing gels compared to those seeded with HFH. The Huh-7.5 hepatoma subline is highly permissive for HCV full length and subgenomic replicons, including those derived from HCV type 1b [72] as well as engineered cell culture tropic HCV type 2a [27], [73]. In contrast, neither human nor chimpanzee-derived natural isolates identified to date can be propagated in Huh-7.5 cells. The presence of neutralizing antibodies in these serum-derived HCV sources complicates use of these strains, and is a possible explanation for the lack of detection following *in vitro* infection of HFH in our study. The lack of antibody in cell culture-derived virus, and the ability to infect with a specific cell culture titer, highlights several advantages of Jc1-based HCV systems. However, some patient sera isolates of HCV have been identified can that replicate in HFH [74]–[76]. Such samples are typically derived from acutely infected patients, prior to the development of antibody. These types of materials are rare and we do not have access to such samples.

We believe that ours is the first report of cell culture tropic virus infection of HFH. We observed association of NS5A with ER membranes in infected cells, which was anticipated from prior IEM studies following *in vitro* infection of Huh7 cells with JFH-1 virus [77]. However, even though human albumin was detectable in the plasma of Huh-7.5 gel-engrafted mice, HCV RNA was below the limit of detection (>10^4^ copies/ml) in plasma harvested at 2 weeks post-engraftment. HFH support HCV particle assembly and release *in vitro* [76], and one would expect the same within the described gel system *in vivo*. Increasing the initial size and cell density of the graft should enhance HCV titers such that they are detectable from plasma samples using qRT-PCR or Huh-7.5-based cell culture. As an adjunct, novel imaging techniques could be considered for real-time monitoring of even low levels of HCV infection within grafts without the need to harvest gel tissue [8]. Intriguingly, we have also been able to detect *in vitro* replication of HCV Jc1 in neonatal, but not adult, hepatocytes [Harding et al., AJP, submitted].

In conclusion, we have characterized a non-injury transplantation strategy in which cultured HFH or human hepatoma cells are engrafted at a subcutaneous site in commercially-available adult SCID/bg mice. Consistent with our hypothesis that inadequate perfusion is a limiting factor for HFH engraftment, higher levels of epithelial cells survived at least 8 weeks post-engraftment within gels that were vascularized by Bcl-2-transduced HUVEC. Following *ex vivo* exposure, both HFH and Huh-7.5 cells sustained HCV Jc1 infection for at least 2 weeks *in vivo*, and was expected to continue for at least 2 months, given the duration of HFH survival within vascularized gels. We believe that our findings represent the first reported success of maintaining HFH on the abdominal wall of immunodeficient mice. This novel system represents a simple small animal model of human primary hepatic epithelial or hepatoma cell engraftment, which should offer new opportunities for studying HCV infections and therapeutics *in vivo*. Future work directed at promoting hepatocellular differentiation and proliferation, and confirmation of Jc1 HCV infection following injections, would serve to further increase the utility of the model.

## Supporting Information

Figure S1 Characterization of neovascularization of passage 1 HFH gels. Tubes were observed forming *in vitro* within 15 hours of HFH gel preparation (A, arrows). A trabecular network of small tubes was observed in gels stained with human-specific CD31 antibody (B), whereas mouse-specific CD31-lined vessels were rarely observed and limited to the gel periphery (C; gel edge is demarcated with arrow). Capillaries were observed in H&E-stained sections (D, arrows) and were determined to be of human origin by virtue of their human CD31 expression (E, arrow). TEM analysis confirmed the presence of morphologically normal capillaries (F, arrow) containing RBC within the gel stroma. Size bars: 100 µm (A), 15 µm (B, C), 10 µm (D, E), and 5 µm (F).
(0.79 MB TIF)

Figure S2 Characterization of neovascularization of passage 1 HFH/Bcl-2-HUVEC gels. At 4 weeks post-engraftment, HFH/Bcl-2-HUVEC gels were 6×4×3 mm3 in size (A) and contained large, multi-layer arterioles (B, arrows) that were not present in matched Bcl-2-HUVEC gels (C). The majority of vessels in the HFH/Bcl-2-HUVEC grafts originated from Bcl-2-HUVEC based on Bcl-2-staining of paraffin-embedded gel tissue (D). The total contribution of donor (human) and host (mouse) vessels within the gel based on human and mouse CD31-staining of unfixed gel sections is shown in E and F, respectively. Size bars: 1 mm (A), 10 µm (B–D), and 15 µm (E, F).
(1.02 MB TIF)

Figure S3 HGF-transduced HUVEC produce bioactive HGF *in vitro*. Conditioned medium (CM; 1∶50 dilution) from various cultures of transduced HUVEC were added to MDCK cells and then representative individual colonies (shown at 0 hr, panels A–D) were tracked for changes in morphology at 24 hours (panels E–H). Note the higher rate of cell proliferation and spreading (“scatter”) at 24 hr following the addition of CM in cultures exposed to Bcl-2/HGF-HUVEC CM (H), compared to media alone (E), HUVEC (F) and Bcl-2-HUVEC (G) CM.
(0.30 MB TIF)
