# Supplementary figures and images for: An Implantable Vascularized Protein Gel Construct That Supports Human Fetal Hepatoblast Survival and Infection by Hepatitis C Virus in Mice
Source: PLoS One. 2010 Apr 1;5(4):e9987. doi: 10.1371/journal.pone.0009987 (PMC2848675; doi:10.1371/journal.pone.0009987)

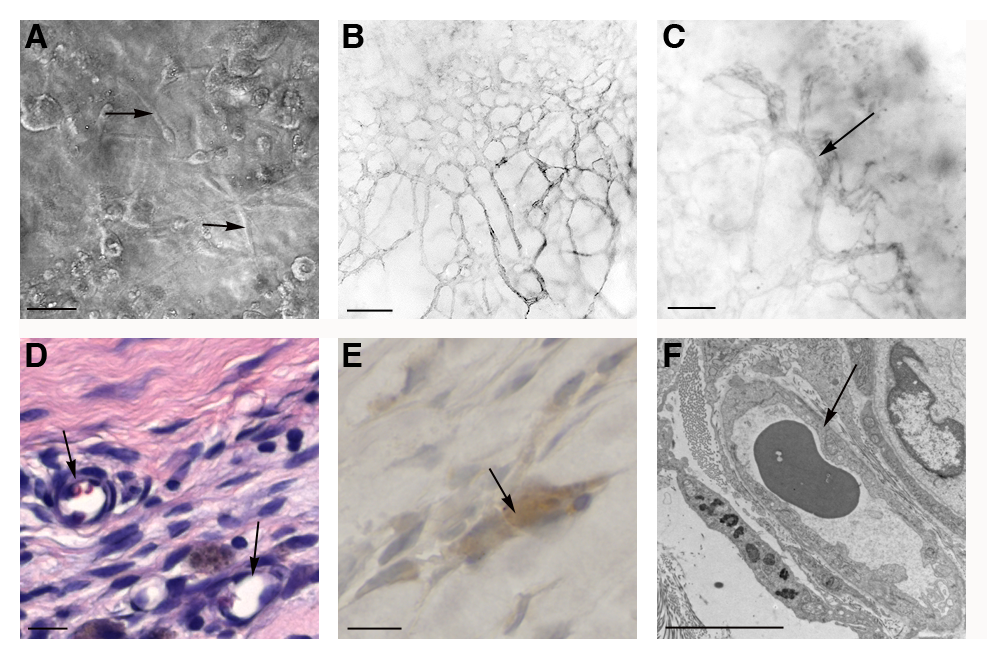

Supplement: Figure S1 — Characterization of neovascularization of passage 1 HFH gels. Tubes were observed forming in vitro within 15 hours of HFH gel preparation (A, arrows). A trabecular network of small tubes was observed in gels stained with human-specific CD31 antibody (B), whereas mouse-specific CD31-lined vessels were rarely observed and limited to the gel periphery (C; gel edge is demarcated with arrow). Capillaries were observed in H&E-stained sections (D, arrows) and were determined to be of human origin by virtue of their human CD31 expression (E, arrow). TEM analysis confirmed the presence of morphologically normal capillaries (F, arrow) containing RBC within the gel stroma. Size bars: 100 µm (A), 15 µm (B, C), 10 µm (D, E), and 5 µm (F). (0.79 MB TIF) [file pone.0009987.s001.tif]

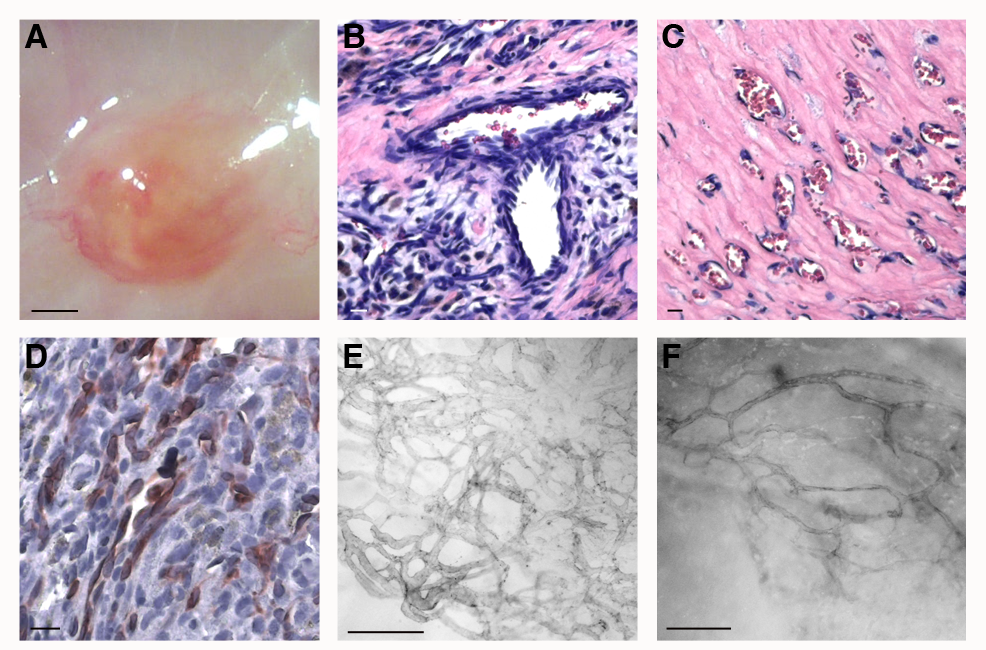

Supplement: Figure S2 — Characterization of neovascularization of passage 1 HFH/Bcl-2-HUVEC gels. At 4 weeks post-engraftment, HFH/Bcl-2-HUVEC gels were 6×4×3 mm3 in size (A) and contained large, multi-layer arterioles (B, arrows) that were not present in matched Bcl-2-HUVEC gels (C). The majority of vessels in the HFH/Bcl-2-HUVEC grafts originated from Bcl-2-HUVEC based on Bcl-2-staining of paraffin-embedded gel tissue (D). The total contribution of donor (human) and host (mouse) vessels within the gel based on human and mouse CD31-staining of unfixed gel sections is shown in E and F, respectively. Size bars: 1 mm (A), 10 µm (B–D), and 15 µm (E, F). (1.02 MB TIF) [file pone.0009987.s002.tif]

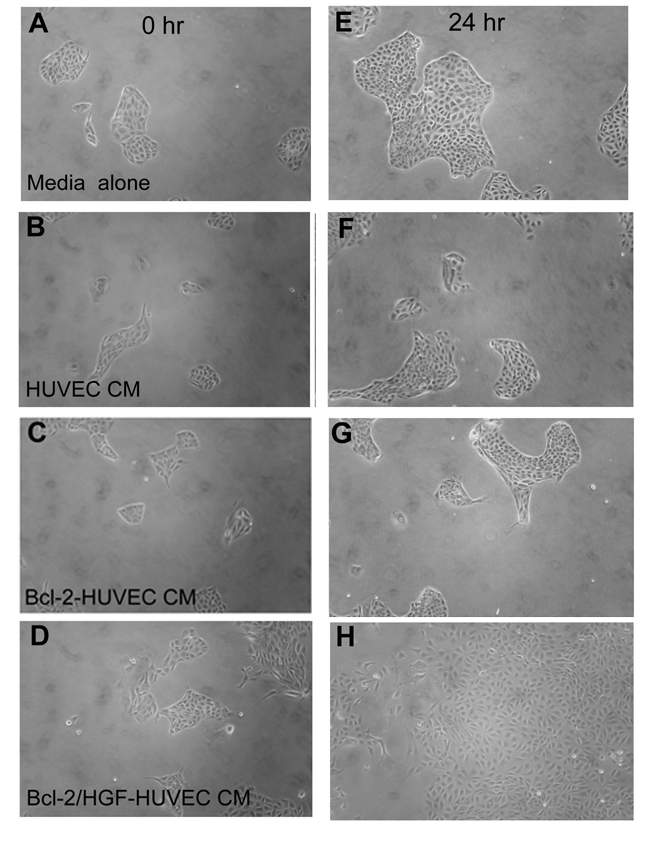

Supplement: Figure S3 — HGF-transduced HUVEC produce bioactive HGF in vitro. Conditioned medium (CM; 1∶50 dilution) from various cultures of transduced HUVEC were added to MDCK cells and then representative individual colonies (shown at 0 hr, panels A–D) were tracked for changes in morphology at 24 hours (panels E–H). Note the higher rate of cell proliferation and spreading (“scatter”) at 24 hr following the addition of CM in cultures exposed to Bcl-2/HGF-HUVEC CM (H), compared to media alone (E), HUVEC (F) and Bcl-2-HUVEC (G) CM. (0.30 MB TIF) [file pone.0009987.s003.tif]
